# Astaxanthin Suppresses Cigarette Smoke-Induced Emphysema through Nrf2 Activation in Mice

**DOI:** 10.3390/md17120673

**Published:** 2019-11-28

**Authors:** Hiroaki Kubo, Kazuhisa Asai, Kazuya Kojima, Arata Sugitani, Yohkoh Kyomoto, Atsuko Okamoto, Kazuhiro Yamada, Naoki Ijiri, Tetsuya Watanabe, Kazuto Hirata, Tomoya Kawaguchi

**Affiliations:** Department of Respiratory Medicine, Graduate School of Medicine, Osaka City University, Osaka 545-8585, Japan

**Keywords:** chronic obstructive pulmonary disease, oxidative stress, astaxanthin, nuclear factor erythroid 2-related factor 2, heme oxygenase-1

## Abstract

Oxidative stress plays an important role in the pathogenesis of chronic obstructive pulmonary disease (COPD). The activation of nuclear factor erythroid 2-related factor 2 (Nrf2) is a key cellular defense mechanism against oxidative stress. Recent studies have shown that astaxanthin protects against oxidative stress via Nrf2. In this study, we investigated the emphysema suppression effect of astaxanthin via Nrf2 in mice. Mice were divided into four groups: control, smoking, astaxanthin, and astaxanthin + smoking. The mice in the smoking and astaxanthin + smoking groups were exposed to cigarette smoke for 12 weeks, and the mice in the astaxanthin and astaxanthin + smoking groups were fed a diet containing astaxanthin. Significantly increased expression levels of Nrf2 and its target gene, heme oxygenase-1 (HO-1), were found in the lung homogenates of astaxanthin-fed mice. The number of inflammatory cells in the bronchoalveolar lavage fluid (BALF) was significantly decreased, and emphysema was significantly suppressed. In conclusion, astaxanthin protects against oxidative stress via Nrf2 and ameliorates cigarette smoke-induced emphysema. Therapy with astaxanthin directed toward activating the Nrf2 pathway has the potential to be a novel preventive and therapeutic strategy for COPD.

## 1. Introduction

Chronic obstructive pulmonary disease (COPD) is caused by the prolonged inhalation of noxious gases, primarily cigarette smoke [1]. Cigarette smoke contains many harmful substances such as oxidants [2]. It has been hypothesized that the etiology of COPD stems from an oxidant–antioxidant imbalance and a protease–antiprotease imbalance. Oxidative stress is an important factor in COPD pathogenesis [3]. Therefore, antioxidant treatment has recently attracted attention in COPD research [4]. Nuclear factor erythroid 2-related factor 2 (Nrf2) is a transcription factor that regulates antioxidant capacity [5]. Nrf2 translocates to the nucleus of the cell and binds to the antioxidant response element (ARE) in response to oxidative stress. Subsequently, Nrf2 initiates the transcription of antioxidant genes and the expression of corresponding proteins. The activation of the Nrf2–ARE signaling pathway is known to be a primary mechanism in the defense against oxidative stress [6]. It has been reported that Nrf2-deficient mice are highly susceptible to cigarette smoke-induced lung injury [7,8]. In addition, the overexpression of Nrf2 was reported to protect against cigarette smoke-induced cell apoptosis [9,10]. These reports suggest that Nrf2 activation protects against the oxidative stress seen in cigarette smoke-induced emphysema.

Astaxanthin is a xanthophyll carotenoid that is widely distributed throughout the world, particularly in marine environments. This compound has potent antioxidant activity, which has been shown to be greater than that of other carotenoids and vitamin E [11,12]. In addition, several studies have reported that astaxanthin activates the Nrf2–ARE signaling pathway as the mechanism for exerting its antioxidant effects [13,14,15,16,17]. However, the suppression of cigarette smoke-induced emphysema by astaxanthin via its antioxidant activity has not yet been reported.

Based on these reports, we hypothesize that astaxanthin enhances Nrf2 expression in the lungs, attenuates oxidative stress, and ameliorates cigarette smoke-induced emphysema. To address this hypothesis, we examined the Nrf2–ARE signaling pathway and the emphysema suppression effect by administering astaxanthin in a murine model of COPD.

## 2. Results

### 2.1. Body Weight Changes in Mice

One mouse from the control group and one mouse from the astaxanthin group were excluded from the analysis due to missing or sample collection failure. Two weeks after starting cigarette smoke exposure, one mouse in the astaxanthin + smoking group died of unknown causes after cigarette smoke exposure.

**Figure 1:**
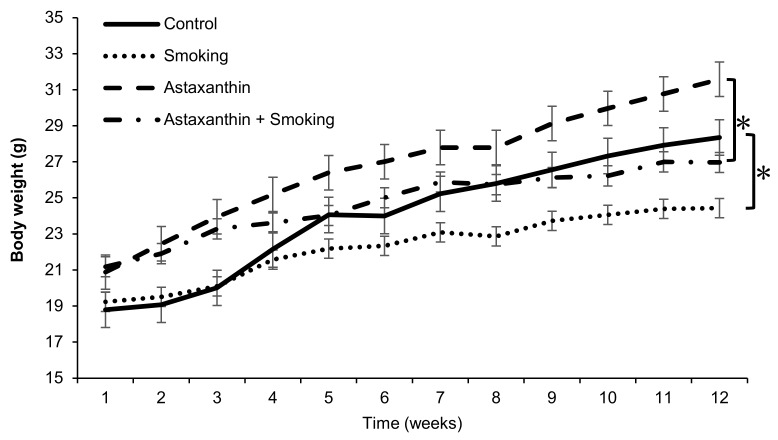
Body weight changes in each group. In both standard diet and astaxanthin-fed groups, smoking exposure significantly decreased weight gain. Although it did not reach statistical significance, the astaxanthin + smoking group gained more weight than the smoking group. Values represent the means ± SD. * *p* < 0.05.

After the 12-week experimental period, the mice in the smoking group showed lower rates of weight gain than those in the control or astaxanthin groups. Although the mice in the astaxanthin + smoking group had higher rates of weight gain than those in the smoking group, the weight gain was less than that of the mice in the control and astaxanthin groups (Figure 1).

### 2.2. Nrf2 and HO-1 Expression Levels Were Increased in Astaxanthin-Fed Mice

**Figure 2:**
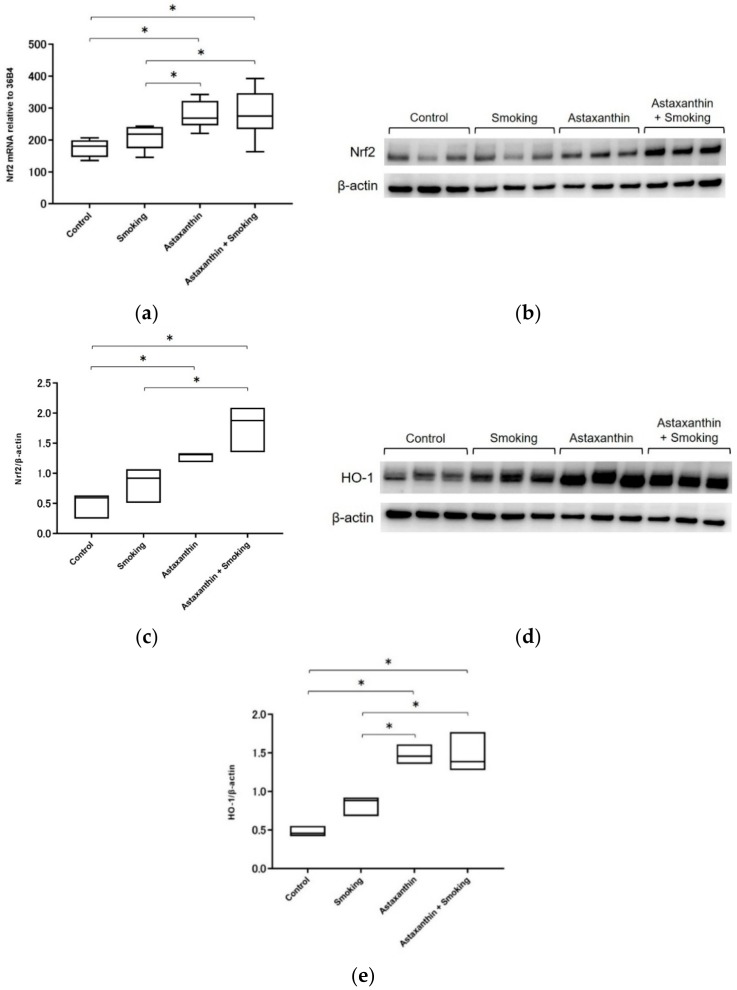
Nrf2 and HO-1 expression in the astaxanthin group was significantly increased compared to that in the control group. Similarly, Nrf2 and HO-1 expression in the astaxanthin + smoking group was significantly increased compared to that in the smoking group. Nrf2 mRNA expression in lung homogenates (**a**). Western blot analysis of Nrf2 in lung homogenates (**b**). The blots were normalized to β-actin and measured by densitometry (**c**). Western blot analysis of HO-1 in lung homogenates (**d**). The blots were normalized to β-actin and measured by densitometry (**e**). * *p* < 0.05.

The Nrf2 mRNA expression levels (as evaluated by real-time PCR) in lung homogenates were significantly increased in the mice in the astaxanthin and astaxanthin + smoking groups compared to those in the control and smoking groups (*p* < 0.05; Figure 2a). No significant difference was observed in Nrf2 mRNA expression levels between the mice in the astaxanthin and astaxanthin + smoking groups. In addition, no significant difference was observed in Nrf2 mRNA expression levels between the mice in the control and smoking groups. 

Nrf2 protein expression levels were assessed by Western blot analysis. Nrf2 protein expression was increased in the mice in the astaxanthin group compared to the control group and in the astaxanthin + smoking groups compared to the smoking group (*p* < 0.05; Figure 2b,c). No significant difference in Nrf2 protein expression was observed between the mice in the control and smoking groups. Similarly, no significant difference in Nrf2 protein expression was observed between the mice in the astaxanthin and the astaxanthin + smoking groups.

To evaluate the Nrf2–ARE signaling pathway, heme oxygenase-1 (HO-1), which is regulated by Nrf2, was also assessed by Western blot analysis. HO-1 protein expression was also increased in the mice in the astaxanthin and astaxanthin + smoking groups compared to that in the control and smoking groups (*p* < 0.05; Figure 2d,e). No significant difference in HO-1 protein expression was observed between the mice in the control and smoking groups. Similarly, no significant difference in HO-1 protein expression was observed between the astaxanthin and astaxanthin + smoking groups.

### 2.3. Astaxanthin Ameliorated Inflammatory Cell Increase in BALF of Cigarette Smoke-Induced COPD

**Figure 3:**
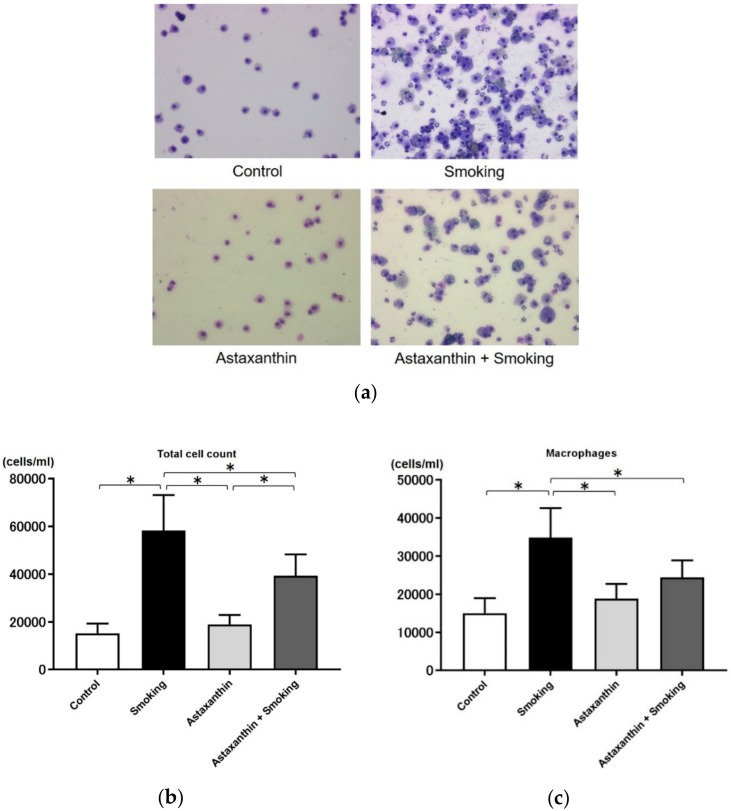

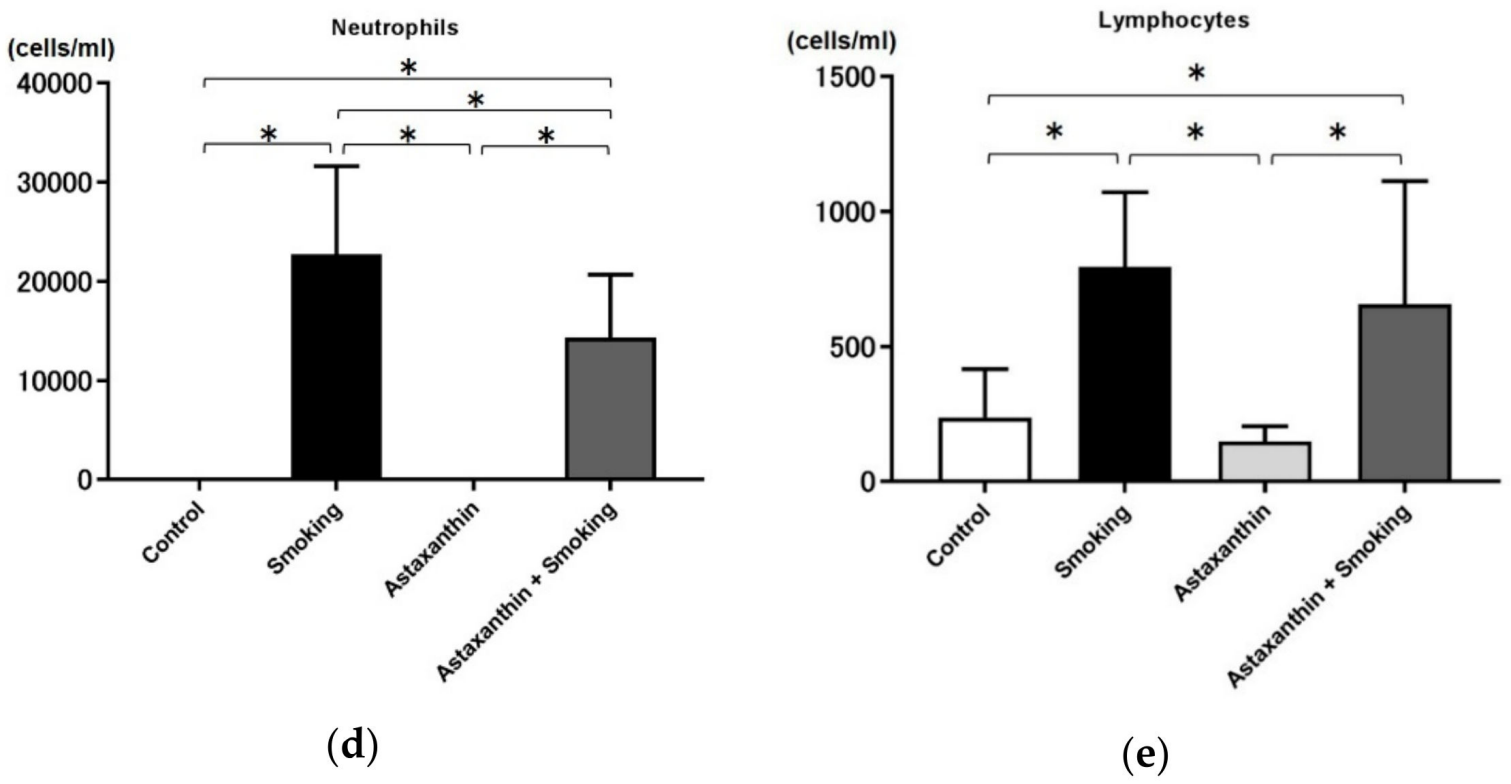
Total cell count and the number of macrophages and neutrophils in the bronchoalveolar lavage fluid (BALF) were significantly lower in the BALF of mice in the astaxanthin + smoking group than in the smoking group, but the number of lymphocytes was not attenuated. Representative images of the BALF from each group are shown at 200× magnification (**a**). Number of total cells (**b**), number of macrophages (**c**), number of neutrophils (**d**), and number of lymphocytes in the BALF (**e**). Values represent the means ± SD. * *p* < 0.05.

A representative image of the bronchoalveolar lavage fluid (BALF) from each group is shown in Figure 3a. To examine the influence of cigarette smoke exposure on BALF and the changes induced by astaxanthin, we enumerated the cell populations and evaluated the number of cells in the BALF. No significant differences in total cell count, the number of macrophages, the number of neutrophils, or the number of lymphocytes in the BALF of mice were observed in the control and astaxanthin groups (Figure 3b–e). The number of neutrophils was significantly higher in the BALF of mice in the smoking and astaxanthin + smoking groups compared to the control and astaxanthin groups due to the effects of smoking exposure (Figure 3d). Total cell count and the number of macrophages and lymphocytes were significantly higher in the BALF of mice in the smoking group compared to the control and astaxanthin groups (Figure 3b,c,e). Total cell count and the number of macrophages and neutrophils were significantly lower in the BALF of mice in the astaxanthin + smoking group compared to the smoking group (*p* < 0.05; Figure 3b–d). No significant difference was observed in the number of lymphocytes in the BALF of mice in the smoking and astaxanthin + smoking groups (Figure 3e).

### 2.4. Astaxanthin Ameliorated Cigarette Smoke-Induced Emphysema

**Figure 4:**
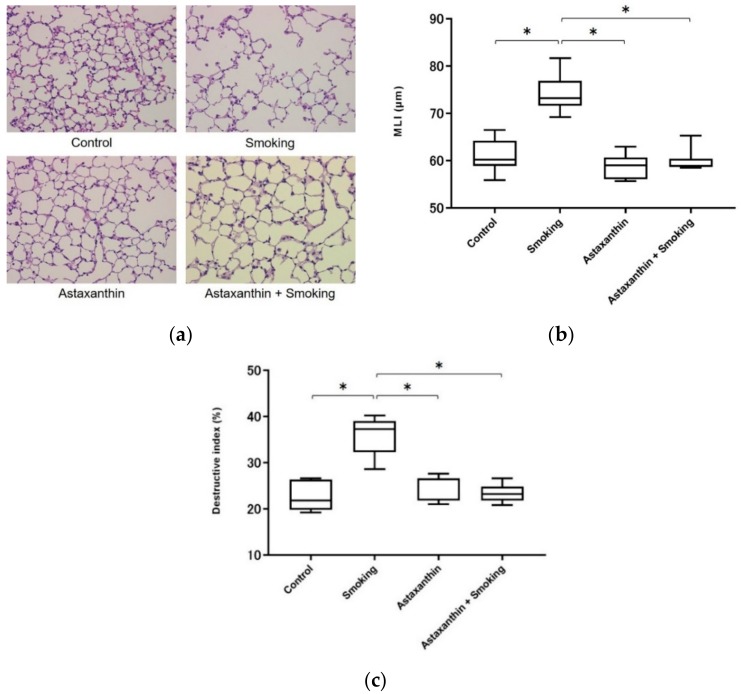
Mean linear intercept (MLI) and destructive index were significantly larger in the smoking group than in the control group. MLI and destructive index were significantly smaller in the astaxanthin + smoking group than in the smoking group. No significant difference was observed in MLI and destructive index between the control, astaxanthin, and astaxanthin + smoking groups. Representative histologic image of lung sections from each group stained with hematoxylin-eosin. Destructed alveolar lesions are indicated by arrows (**a**). MLI data (**b**). Destructive index data (**c**). * *p* < 0.05.

A representative histologic image of the lung from each group is shown in Figure 4a. The exposure to cigarette smoke for 12 weeks resulted in the development of pulmonary emphysema in the mice in the smoking group. The lung tissues from the astaxanthin + smoking group showed lower alveolar destruction than the lung tissues of the mice in the smoking group. Mean linear intercept (MLI) was significantly larger in the mice in the smoking group than in the control group, and MLI for the mice in the astaxanthin + smoking group was significantly lower than that reported for the mice in the smoking group (*p* < 0.05; Figure 4b). No significant difference was observed in MLI between the mice in the control, astaxanthin, and astaxanthin + smoking groups. Moreover, the destructive index was significantly larger in the mice in the smoking group than in the control or astaxanthin groups, and the destructive index was significantly lower in the mice in the astaxanthin + smoking group than in the smoking group (*p* < 0.05; Figure 4c). No significant difference was observed in the destructive index between the mice in the control, astaxanthin, and astaxanthin + smoking groups.

## 3. Discussion

In this study, we showed that astaxanthin increased Nrf2 and HO-1 expression in lung tissue and suppressed cigarette smoke-induced emphysema in mice. Our results indicate that the ingestion of astaxanthin suppresses cigarette smoke-induced inflammatory cell infiltration in the BALF and emphysema by activating the Nrf2–ARE signaling pathway in the lungs in a murine model of COPD.

COPD is the third leading cause of death in the world [18]. However, current therapies for COPD provide only limited benefit and fail to halt progression. Therefore, the development of new prevention and treatment strategies for COPD is necessary. Cigarette smoke is the primary cause of COPD, and it contains many oxidants [2]. An insufficient antioxidant capacity is related to COPD pathogenesis [19]. An excess of oxidants has been reported to induce emphysema through epithelial cell apoptosis [20]. In recent decades, oxidative stress has been recognized as a key factor responsible for the pathogenesis of COPD [21].

Previously, we reported that N-acetylcysteine significantly suppressed cigarette smoke extract-induced apoptosis of airway epithelial cells [22]. This result suggests that antioxidants such as N-acetylcysteine may suppress cigarette smoke-induced apoptosis and emphysema in models of COPD. Epidemiologic evidence also supports the potential beneficial effects of an antioxidant-rich diet on pulmonary function and COPD risk [23]. Antioxidant therapy or supplemental treatment with an external antioxidant to neutralize excess oxidants may have great therapeutic potential in COPD [24].

Nrf2 is a transcription factor involved in the regulation of various antioxidants. In response to oxidative stress, Nrf2 translocates to the nucleus and binds the ARE of target genes involved in an antioxidant response. Subsequently, Nrf2 initiates the transcription and expression of antioxidant proteins. Then, antioxidant proteins induced by Nrf2, such as HO-1, protect against oxidative stress [25]. Nrf2 is expressed in various organs including the lung. Nrf2-deficient mice show reduced activity of antioxidant enzymes, are susceptible to cigarette smoke, and develop severe lung emphysema [7,8]. Moreover, increased Nrf2 activation was shown to attenuate the oxidative stress of cigarette smoke and protect cells from apoptosis induced by oxidative stress [9,10]. We previously showed that Nrf2 expression was significantly reduced in the airway epithelial cells of COPD patients [22]. In addition, other studies indicate the relationship of Nrf2 polymorphisms and airflow limitations in smokers [26,27]. Recently, we reported that a polymorphism of the Nrf2 gene contributed to the progression of lung emphysema in smokers [28]. From these findings, Nrf2 is considered to be prominently involved in the pathogenesis of COPD.

To our knowledge, there is no report that astaxanthin is related to the prevention of COPD. Astaxanthin, a carotenoid xanthophyll, is a natural reddish-orange pigment widely present in nature. Astaxanthin is especially abundant in marine organisms such as shrimp, crab, salmon, krill, and algae. Since ancient times, these crustaceans and fishes have been eaten by humans. Astaxanthin ingestion is safe, and pure astaxanthin was approved as a dietary supplement by the Food and Drug Administration in the United States in 1999 [29]. Recently, the technology for mass-producing astaxanthin by culturing *Haematococcus pluvialis* was developed, and it has become simple to obtain a large amount of astaxanthin. In fact, astaxanthin is widely used in cosmetics because it has been reported to protect the skin from ultraviolet rays and help maintain healthy skin [30]. Astaxanthin has attracted attention due to its strong antioxidant properties, and there have been many reports focusing on its antioxidant activity. Astaxanthin has been shown to protect various cells from oxidative stress in vitro [31,32,33,34] and to protect the brain, eyes, salivary glands, skeletal muscle, liver, kidney, and lungs from oxidative stress in vivo [12,13,14,15,16,17,35,36,37,38,39]. These results indicate that astaxanthin is distributed throughout the body and has systemic effects. Moreover, previous studies have reported that astaxanthin enhances Nrf2 expression in various organs including the lungs [13,14,15,16,17]. Additionally, some studies have investigated the pathway of Nrf2 activation by astaxanthin. Astaxanthin facilitates the dissociation and nuclear translocation of Nrf2 through activation of the PI3K/Akt and ERK signaling pathways [40,41].

In our study, Nrf2 expression in the lungs was slightly higher in the smoking group than in the control group; however, no significant difference was observed. Cigarette smoke-induced oxidants were potentially stronger than the protective effect of the antioxidants in the smoking group, which may have caused emphysema. In contrast, Nrf2 expression was significantly increased in the astaxanthin + smoking group compared to the smoking group. Therefore, the antioxidants may have exerted stronger effects than the cigarette smoke-induced oxidants and suppressed the development of emphysema in the astaxanthin + smoking group. 

Previous studies have reported that emphysema was suppressed by administering antioxidant substances to mice. Hydrogen has been found to be a strong antioxidant, and administration of hydrogen-rich water was reported to attenuate cigarette smoke-induced lung damage and reduce the MLI in senescence marker protein-30 knockout mice [42]. 2-Cyano-3,12-dioxooleana-1,9-dien-28-oic acid (CDDO) has also been reported to have an Nrf2 activation effect. CDDO-imidazolide administered during a period of cigarette smoke inhalation was shown to suppress pulmonary emphysema via Nrf2 in mice [43]. These reports support our results.

We showed that astaxanthin inhibited cigarette smoke-induced inflammatory cell infiltration in BALF. Although Nrf2 suppresses inflammation as a secondary consequence of its antioxidant effect, astaxanthin has been also reported to directly suppress inflammation [44,45]. The suppression of inflammatory cell infiltration in BALF may also be related to this property of astaxanthin.

Oxidative stress caused by cigarette smoke has been reported to persist long after smoking cessation [46]. Prolonged oxidative stress is a primary factor in the enhancement of both airway and systemic inflammation in COPD patients and is known to play an important role in the development of COPD and its comorbidities [47]. Therefore, it may be possible to suppress persistent oxidative stress and inflammation by the ingestion of astaxanthin even after smoking cessation; it may also be possible to treat COPD and its comorbidities with a single therapeutic agent. Ingestion of astaxanthin has been proven to be safe, it is widely used in beauty products, and mass production methods have been established. Therefore, astaxanthin may have the potential to serve as a therapeutic agent or a supplement for COPD in the near future.

This study has some limitations. First, the concentrations of astaxanthin in the blood of mice were not determined and bioavailability is unknown. Second, the concentration of astaxanthin (0.02% w/w) in the diet was taken from a previous study [48]. In addition, 50 mg/kg of astaxanthin was reported to be effective in mice [37]. Therefore, we decided to use the diet to contain 0.02% (w/w) astaxanthin. However, the optimal effective concentration of astaxanthin is unknown. Further research is needed to clarify these points.

## 4. Materials and Methods 

### 4.1. Experimental Animals

C57BL/6 mice (male, four weeks old, 18–20 g) were obtained from Japan SLC (Shizuoka, Japan) and kept under pathogen-free conditions. The mice were maintained at a controlled temperature of 23 °C ± 2 °C under a 12:12 h light–dark cycle with free access to water. The mice were divided into four groups as follows: (1) control (n = 8), (2) smoking (n = 8), (3) astaxanthin (n = 8), (4) astaxanthin + smoking (n = 8). All mice were acclimatized to the environment for one week. The mice in the astaxanthin and astaxanthin + smoking groups were fed a diet containing astaxanthin (FUJIFILM ASTAXANTHIN 10O; FUJIFILM Corporation, Tokyo, Japan). We prepared the diet to contain 0.02% (w/w) astaxanthin; the concentration of astaxanthin was measured by using high-performance liquid chromatography after enzymatic degradation of fatty acid ester form of astaxanthin to free form of astaxanthin. The actual concentration of the diet was determined to be 0.0158% (w/w). The mice in the control and smoking groups were fed a standard diet. All experimental protocols were approved by the Ethics Committee of the Institutional Animal Care and Use of Osaka City University Graduate School of Medicine (17023, 6/11/2017). Animal experiments were conducted in accordance with the Regulations on Animal Experiments in Osaka City University following the Guidelines for Proper Conduct of Animal Experiments in Japan.

### 4.2. Experimental Model of Cigarette Smoke-Induced COPD

The mice in the smoking and astaxanthin + smoking groups were exposed to cigarette smoking (18 cigarettes/day) for 60 min once daily, 5 days per week. Commercially available Peace^®^ nonfilter cigarettes (2.3 mg nicotine and 28 mg tar/cigarette; Japan Tobacco, Tokyo, Japan) and a cigarette smoke generator model SG-300 for small animals (Shibata Scientific Technology, Tokyo, Japan) were used for the cigarette smoke exposure. The mice in the control and astaxanthin groups were exposed to fresh air. Cigarette smoke and fresh air exposure was performed for 12 weeks.

### 4.3. Treatments and Preparation for Evaluation

At the end of the 12-week experimental period, all mice were sacrificed under deep anesthesia. The mice were tracheotomized and cannulated, and bronchoalveolar lavage (BAL) was performed three times with 0.5 mL phosphate-buffered saline for sampling of the BALF. After BAL, the right lung of each mouse was carefully excised. The right lower lobe was instantly soaked in RNAlater (Invitrogen by Thermo Fisher Scientific, Waltham, MA, USA) for mRNA expression analysis. The other right lobe was immediately frozen in liquid nitrogen for protein expression analysis. The left lung was excised and immediately soaked in 10% formalin for further histological analysis.

### 4.4. Nrf2 mRNA Expression Analysis

The right lower lobe was homogenized in RLT lysis buffer (Qiagen NV, Venlo, Netherlands). RNeasy mini kit (Qiagen NV, Venlo, Netherlands) was used for the extraction of total RNA. Complementary DNA (cDNA) was obtained by reverse transcription of the mRNA with the Ready-to-Go T-primed first-strand kit (GE Healthcare, Little Chalfont, UK). The cDNA was used in a real-time quantitative PCR reaction in an Applied Biosystems 7500 real-time PCR system (Thermo Fisher Scientific, Waltham, MA, USA) using TaqMan gene expression assays for Nrf2 (Mm00477784_m1). The housekeeping gene *36B4* (Mm00725448_s1) was used for the normalization of Nrf2 mRNA as previously described [49].

### 4.5. Western Blot Analysis

The right lung other than the lower lobe was used for Western blot analysis. Approximately 30 mg of the lung sample was soaked in 300 µL of radioimmunoprecipitation assay (RIPA) buffer (Beyotime Biotechnology, Shanghai, China) supplemented with the protease inhibitor phenylmethanesulfonyl fluoride (Beyotime Biotechnology, Shanghai, China) and Protease Inhibitor Cocktail (Cell Signaling Technology Japan, Tokyo, Japan). After the lung samples were homogenized in RIPA buffer, the samples were placed on ice for 5 min and centrifuged at 11,800× *g* and 4 °C for 4 min. The supernatant was collected, and the protein concentration was determined with the colorimetric bicinchoninic acid protein assay kit (Pierce, Waltham, MA, USA) according to the manufacturer’s instructions. The supernatant was subjected to sodium dodecyl sulfate polyacrylamide gel electrophoresis with Mini-PROTEAN TGX Precast Protein Gels (4561023, Bio-Rad, Hercules, California, USA). Next, the separated bands on the gel were transferred onto polyvinylidene fluoride membranes. The membranes were then incubated with primary anti-Nrf2 antibody (1:500; ab137550, Abcam, Cambridge, UK), anti-HO-1 antibody (1:250; ab13248, Abcam), or anti-β-actin antibody (1:1000; ab8227, Abcam) at 4 °C overnight. The next day, the membranes were incubated with the corresponding secondary antibodies for 2 h at 25 °C. After washing the membranes three times, SuperSignal West Dura Extended Duration Substrate (Thermo Fisher Scientific, Waltham, MA, USA) was used for detection. Western blot signals were acquired with a Fuji LAS-4000 fluorescence imager (Fujifilm Corporation, Tokyo, Japan). The target protein levels were normalized to β-actin.

### 4.6. Bronchoalveolar Lavage Fluid Analysis

Each BALF sample was centrifuged at 1200× *g* and 4 °C for 10 min, and the supernatant was collected. The cell pellet was resuspended in 1 mL of phosphate-buffered saline and applied to cytospin columns in a Shandon Cytospin 3 centrifuge (Shandon Scientific Co., London, England), and the cytospin protocol was followed. The slides sprayed with the cells were stained with Diff-Quick (Sysmex, Kobe, Japan), and the enumeration of cells and the differential cell counts (macrophages, neutrophils, and lymphocytes) were performed in a blind manner.

### 4.7. Quantitative Evaluation of Lung Emphysema

The left lung was fixed with 10% formalin for 24–48 h at positive pressure (25 cm H_2_O) and subjected to histological analysis. Three-micrometer-thick slices were stained with hematoxylin and eosin for the analysis of the level of airspace size in the lung. Emphysema was evaluated by determining the MLI as previously described [50]. Moreover, destruction was evaluated by determining the destructive index as previously described [51].

### 4.8. Statistical Analysis

Data are expressed as the mean ± standard deviation. For multiple-group comparisons, differences were evaluated using one-way ANOVA followed by Tukey’s multiple comparison test. Statistical significance was considered at *p* < 0.05. All statistical analyses were performed using GraphPad Prism 7.04 (GraphPad Software, San Diego, CA, USA).

## 5. Conclusions

COPD is associated with an excessive oxidant burden; therefore, the rationale for exploring antioxidant therapies in COPD is clear. Astaxanthin increases Nrf2 and HO-1 expression in the lung and suppresses cigarette smoke-induced emphysema in mice. Therapy directed toward activating the Nrf2–ARE pathway, such as the use of astaxanthin, may be a novel preventive and therapeutic strategy for attenuating oxidative stress in the pathogenesis of COPD.
